# Variable interaction specificity and symbiont performance in Panamanian *Trachymyrmex* and *Sericomyrmex* fungus-growing ants

**DOI:** 10.1186/s12862-014-0244-6

**Published:** 2014-12-04

**Authors:** Henrik H De Fine Licht, Jacobus J Boomsma

**Affiliations:** Centre for Social Evolution, Department of Biology, University of Copenhagen, Universitetsparken 15, DK-2100 Copenhagen, Denmark; Present address: Section for Organismal Biology, Department of Plant and Environmental Sciences, University of Copenhagen, Thorvaldsensvej 40, DK-1871 Frederiksberg, Denmark

**Keywords:** AZCL insoluble chromogenic substrates, Attini, *Leucoagaricus*

## Abstract

**Background:**

Cooperative benefits of mutualistic interactions are affected by genetic variation among the interacting partners, which may have consequences for interaction-specificities across guilds of sympatric species with similar mutualistic life histories. The gardens of fungus-growing (attine) ants produce carbohydrate active enzymes that degrade plant material collected by the ants and offer them food in exchange. The spectrum of these enzyme activities is an important symbiont service to the host but may vary among cultivar genotypes. The sympatric occurrence of several *Trachymyrmex* and *Sericomyrmex* higher attine ants in Gamboa, Panama provided the opportunity to do a quantitative study of species-level interaction-specificity.

**Results:**

We genotyped the ants for Cytochrome Oxidase and their *Leucoagaricus* fungal cultivars for ITS rDNA. Combined with activity measurements for 12 carbohydrate active enzymes, these data allowed us to test whether garden enzyme activity was affected by fungal strain, farming ants or combinations of the two. We detected two cryptic ant species, raising ant species number from four to six, and we show that the 38 sampled colonies reared a total of seven fungal haplotypes that were different enough to represent separate *Leucoagaricus* species. The *Sericomyrmex* species and one of the *Trachymyrmex* species reared the same fungal cultivar in all sampled colonies, but the remaining four *Trachymyrmex* species largely shared the other cultivars. Fungal enzyme activity spectra were significantly affected by both cultivar species and farming ant species, and more so for certain ant-cultivar combinations than others. However, relative changes in activity of single enzymes only depended on cultivar genotype and not on the ant species farming a cultivar.

**Conclusions:**

Ant cultivar symbiont-specificity varied from almost full symbiont sharing to one-to-one specialization, suggesting that trade-offs between enzyme activity spectra and life-history traits such as desiccation tolerance, disease susceptibility and temperature sensitivity may apply in some combinations but not in others. We hypothesize that this may be related to ecological specialization in general, but this awaits further testing. Our finding of both cryptic ant species and extensive cultivar diversity underlines the importance of identifying all species-level variation before embarking on estimates of interaction specificity.

**Electronic supplementary material:**

The online version of this article (doi:10.1186/s12862-014-0244-6) contains supplementary material, which is available to authorized users.

## Background

Considerable progress has been made in understanding the origins, elaborations and occasional collapse of obligate symbiotic mutualisms [1-5]. One of the most crucial aspects for understanding the evolutionary stability of such interactions is their degree of uni- or bilateral specialization [6,7] and integrative complementarity [8,9]. Several recent models have addressed questions of this kind, either emphasizing the dynamics of partner variation in one-to-one interactions [10,11], or that hosts will settle for mixed communities of symbionts dominated by an unambiguous mutualist [12-14]. Empirical studies have also yielded surprises, for example showing that several Central American figs have multiple pollinating wasps that are morphologically indistinguishable [15], and that mountain pine beetles (*Dendroctonus ponderosae*) cultivate multiple fungal species segregating in distinct populations with variable recombination rates [16]. In general, however, studies of this kind are constrained by the need for local biodiversity to be high enough to obtain sufficient statistical power, and by the regions where such species richness is present having many cryptic species so that interaction-specificity will be underestimated. This underlines that it is of crucial importance that empirical studies use genetic markers to establish the true species-level diversity of local guilds of hosts and symbionts before embarking on analyses of interaction specificity. Here we document variation in interaction specificity and genetic diversity in a 50-million-year-old obligate nutritional mutualism between ants and fungi and measure functional enzyme activity variation across sympatric host and symbiont species.

The fungus-growing attine ants comprise >230 extant species, which all obligately cultivate fungus gardens for food while providing them with scavenged or actively harvested plant material as manure. Fungus gardens consist of a single basidiomycete fungal strain that is cultivated in monoculture, but also contains bacteria and yeasts in variable prevalences [17-20]. As a rule of thumb, the attine ants show a large degree of co-phylogenetic congruence with their fungal cultivars at basal levels, but they often share cultivars at the ant-genus level, which has been described as a form of 'diffuse’ coevolution [21-23]. The phylogenetically derived higher-attine genera *Trachymyrmex, Sericomyrmex, Acromyrmex* and *Atta* cultivate specialized *Leucocoprinaceous* fungi that have only been found in association with attine ants [24]. Virgin queens normally carry a fragment of mycelium from her maternal fungus garden as inoculum when founding new colonies [25,26], but this vertically transmission routine may be punctuated by occasional events of secondary horizontal exchange [27]. The ants normally suppress tendencies of fungus gardens to reproduce sexually via mushrooms, and as far as these have been reported it remains unclear whether they can produce viable haploid spores under natural conditions [28]. Indications for some admixture and possible recombination have been found [29], but evidence that this relates to meiotic sexual events is lacking.

The ca. 45 extant species of *Atta* and *Acromyrmex* leaf-cutting ants all appear to cultivate haplotypes of a single species *L. gongylophorus* [29,30], but the *Leucoagaricus* symbionts of *Trachymyrmex* and *Sericomyrmex* have higher genetic diversity [31]. However, this insight is based on a single study of *T. septentrionalis* cultivating four different species-level lineages of fungus towards the northern distribution limit of the attine ants [32]. Species-level interaction specificity (sensu [33]) in richer tropical communities has remained unstudied, so it remains unknown whether: 1. Sympatric ant species belonging to the same ecological “guild” always associate with multiple symbiont species or occasionally cultivate a single symbiont in spite of alternatives being available, and 2. Sympatry implies that non-specialized hosts always share all available symbiont species. The objective of our study was to assess interaction specificity of *Trachymyrmex* and *Sericomyrmex* fungus-growing ants living sympatrically in a seasonal lowland rainforest ecosystem in Panama, by genotyping both the ants and their fungus gardens and measuring the activity of plant cell-wall degrading enzymes immediately upon collection.

As their name implies, leaf-cutting ants primarily forage for fresh leaves, whereas *Trachymyrmex* and *Sericomyrmex* species collect a much more diverse spectrum of freshly shed flowers, thin fallen leaf fragments, minor twigs, caterpillar feces and seeds [25,34]. Fungus-growing ant foraging profiles vary in space and time, but have a substantial species-specific variance component [34,35] that will affect fungus garden enzyme activity because carbohydrate-degrading enzymes are induced rather than constitutively produced [36]. By focusing our sampling on a single geographical location and specific time of the year we ensured as much as possible that foraging spectra reflected natural local niche differentiation. Differences in fungus garden enzyme activity among cultivar genotypes were thus likely to reflect performance differences of direct mutualistic relevance [37].

## Methods

Fungus-growing ants were collected as entire colonies with fungus gardens in May 2008 in Parque National Soberanía, Panama (the Gamboa area and forest along Pipeline Road): ten colonies of *T. cornetzi* (Forel), nine colonies of *T.* sp. 3, nine colonies of *T. zeteki* (Weber), and ten colonies of *Sericomyrmex amabilis* (Wheeler), giving a total of 38 colonies that were brought to the Smithsonian Tropical Research Institute (STRI) laboratory in Gamboa, Panama. *Trachymyrmex* sp. 3 (“black-head”) is a known undescribed species that occurs sympatrically with *T. cornetzi* in its investigated range in Panama [38]. These *Trachymyrmex* and *Sericomyrmex* species were previously shown to have large randomly mating populations at our sampling site (Parque National Soberanía) [39], so the probability that we sampled colonies with recent common descent was negligible.

### Fungal cultivar and host ant genotyping

Fungal DNA was extracted by placing small tufts of mycelium from alcohol (96%) preserved fungus garden material in 625 μl of a 20% Chelex® 100 resin (Sigma-Aldrich, cat. no. 95621) solution with 2 μl Proteinase K (10 mg/mL) and incubated at 60°C for 90 min. followed by 99°C for 15 min. The primers ITS1 and ITS4 [40] were used to amplify the internal transcribed spacer (ITS) region in the nuclear ribosomal RNA gene using one cycle of 95°C for 2 min, followed by 25 cycles of 95°C 30 sec, 54°C for 30 sec, and 72°C for 30 sec, and ending with one cycle of 72°C for 7 min min. PCR products were purified and sequenced by Eurofins MWG-Operon, Ebersberg, Germany [GenBank: KJ855926-KJ855963]. Because DNA was extracted directly from fungus garden material all cultivar sequences were BLAST searched against GenBank sequences to verify their leucocoprinaceous identity.

A single worker per colony was used for DNA extraction. Head, gaster, and legs were removed from each specimen and the thorax crushed between forceps and placed in a 20% Chelex® 100 resin solution and DNA extracted similar to the fungal material. A fragment covering the partial Cytochrome Oxidase 1, tRNA, and Cytochrome Oxidase 2 regions was amplified by a combination of the universal arthropod primers: *George*, *Marilyn*, *Ben* and *Jerry* as previously described [41-43], and sequenced by Eurofins MWG-Operon, Ebersberg, Germany [GenBank: KJ855888-KJ855925].

### Phylogenetic analysis

The 38 partial CO1, tRNA, and CO2 fungus-growing ant sequences were used to construct a 549 bp alignment using ClustalW [44]. The alignment was manually inspected and the sequences of *Atta cephalotes* [GenBank:AF016016] and *Acromyrmex octospinosus* [GenBank:AF016014] included to specify the phylogenetic relationship with leaf-cutting ant genera. ModelTest was used to determine the DNA substitution model (GTR + i + G) and evaluated with AIC scores as implemented in Topali [45]. Maximum likelihood phylogenetic estimation was performed using RaxML [46], with identical sequences removed prior to analysis with 500 bootstrap replicates. Bayesian analyses were performed using MrBayes ver 3.1 [47] and executed from within Topali with default settings. The 38 partial ITS sequences of cultivars were analyzed in a similar way as the ant sequences. A 740 bp alignment including the ITS sequences from a cultivar of *Atta cephalotes* [GenBank:KF571985] and *Acromyrmex octospinosus* [GenBank:KF57994] were constructed with ClustalW and manually inspected. ModelTest determined the DNA substitution model to GTR + G and maximum likelihood phylogenetic estimation was performed using RaxML, after which Bayesian analyses were performed as described above.

We also constructed ant and fungal haplotype networks from the CO and ITS sequences, respectively using phylogenetic median-joining network analysis [48] as implemented in the Free Phylogenetic Network Software [49] and TCS v. 1.21 [50]. The median-joining method first constructs the minimum spanning networks before adding a few consensus sequences that function as median vectors in order to arrive at the most parsimonious networks [48]. The TCS program collapses identical sequences into haplotypes, calculates haplotype frequencies and connects them into a network by calculating an absolute pairwise distance matrix and implementing a statistical parsimony approach that estimates genealogical relationships between mutational differences at a probability (0.90) of parsimony [51].

### Statistical analysis of host-specificity

Analysis of Molecular Variance (AMOVA) as implemented in Arlequin ver. 3.11 [52] was used to partition fungal ITS sequence variation among isolates at three hierarchical levels: between host genera (*Trachymyrmex* or *Sericomyrmex*), between host species within genera (*T. zeteki, T.* sp*. 3, T. cornetzi* sp. 1, *T. cornetzi* sp. 2, and *T. cornetzi* sp. 3), and between colonies within species. Uncorrected pairwise ITS distances were used as a measure of genetic distance between fungal haplotypes and significance was assessed by 10,100 random permutations. Because *S. amabilis* and *T.* sp. 3 each cultivated a single distinct fungal haplotype, A and B respectively, the AMOVA was also performed after excluding these two species and thus only containing *Trachymyrmex* species cultivating more than a single species of symbiont.

To validate the AMOVA results, we constructed a contingency table with columns representing ant species and rows fungal ITS haplotypes, and each cell containing the observed number of ant-fungal combination, so that possible patterns of specificity of randomness (independence) could be assessed with Fisher’s exact test as implemented in R [53]. We performed the same two tests as in the AMOVAs by first considering the entire data set and after that only the data for the four ant species (*T. zeteki, T.* sp*. 3, T. cornetzi* sp. 1, *T. cornetzi* sp. 2, and *T. cornetzi* sp. 3) that cultivated more than a single fungal haplotype.

### Enzyme activity measurements

Upon collection, the fungus gardens were immediately measured for enzyme activity before any food items were administered. Visible ants, larvae, pupae and eggs were removed before total proteins were extracted by grinding 120 mg fresh garden material with a sterile pestle in 1.5 mL Eppendorf tubes containing 500 μl 50 mM Tris pH 7.0. Extracts were centrifuged at 12,400 g for 15 min and the supernatants containing crude total protein extracts were immediately used in enzyme activity assays. Enzyme activity was assayed with Azurine-Crosslinked (AZCL) polysaccharides as previously described [36]. Briefly, an agarose medium of 1% agarose, 23 mM phosphoric acid, 23 mM acetic acid and 23 mM boric acid was heated until the agarose was melted and then cooled to 65°C when 0.1% weight/volume AZCL substrate was added and the medium poured into Petri dishes. Wells were made in the solidified agarose plates with a cut off pipette tip to give a constant diameter of 4 mm before 15 μl of enzyme supernatant was placed in each well. The plates were incubated for 22 hours at 21°C and the area of the blue halo surrounding the well was photographed and measured using the software program ImageJ ver. 1.37 [54].

AZCL-polysaccharides are highly purified polysaccharides, which are dyed with azurine-blue and cross-linked to form a water insoluble chromogenic substrate assay (AZCL, Megazyme^©^). Enzymes present in the protein extracts diffuse into the assay media and in the event of a positive reaction the hydrolysis of AZCL-polysaccharides releases dyed water-soluble fragments at a rate that is proportional to enzyme activity [55]. Measuring the area of blue-coloration on the assay plates is therefore a quantitative measure of enzyme activity against the polysaccharide substrate used [36,56,57]. AZCL plate assays do not provide absolute enzyme activities and are less sensitive than laboratory-based photometric assays standardized to protein content. However, field measurements have high reproducibility and are suitable for larger-scale comparisons of enzyme activity spectra with natural, rather than laboratory, substrates [37]. We used 12 different AZCL-polysaccharides to test for enzyme activity that cleave the polysaccharide chain of stored starch and proteins inside the plant cells and the pectins, celluloses and xylans associated with the plant cell walls (Table 1). Analysis of variance (ANOVA) with type of enzyme, cultivar haplotype, ant species, and their interaction terms as explanatory variables were used to analyze enzyme activity using R [53]. The enzyme activity measurements are provided as supplementary dataset [see Additional file 1].

**Table 1:** **The 12 specific types of enzyme activity measured with insoluble chromogenic AZCL substrates**

**Substrate**	**Enzyme**
**Starch**	
AZCL-Amylose	α-amylase
**Protein**	
AZCL-Casein	*endo*-protease
AZCL-Collagen	*endo*-protease
**Pectin**	
AZCL-Debr. Arabinan	*endo*-α-1,5-arabinase
AZCL-Rhamnogalacturonan	Rhamnogalacturonanase
AZCL-Galactomannan	*endo*-β-1,4-mannanase
AZCL-Galactan	*endo*-β-1,4-galactanase
**Cellulose**	
AZCL-HE-Cellulose	Cellulase (*endo*-β-1,4-glucanase)
AZCL-Barley β-Glucan	Cellulase (*endo*-β-1,3(4)-glucanase)
AZCL-Xyloglucan	*endo*-β-1,4-xyloglucanase
**Cross-linking Glycans**	
AZCL-Xylan	*endo*-β-1,4-xylanase
AZCL-Arabinoxylan	*endo*-β-1,4-xylanase

AZCL = Azurine cross-linked polysaccharides (Megazyme^©^, Bray, Ireland).

## Results

**Figure 1:**
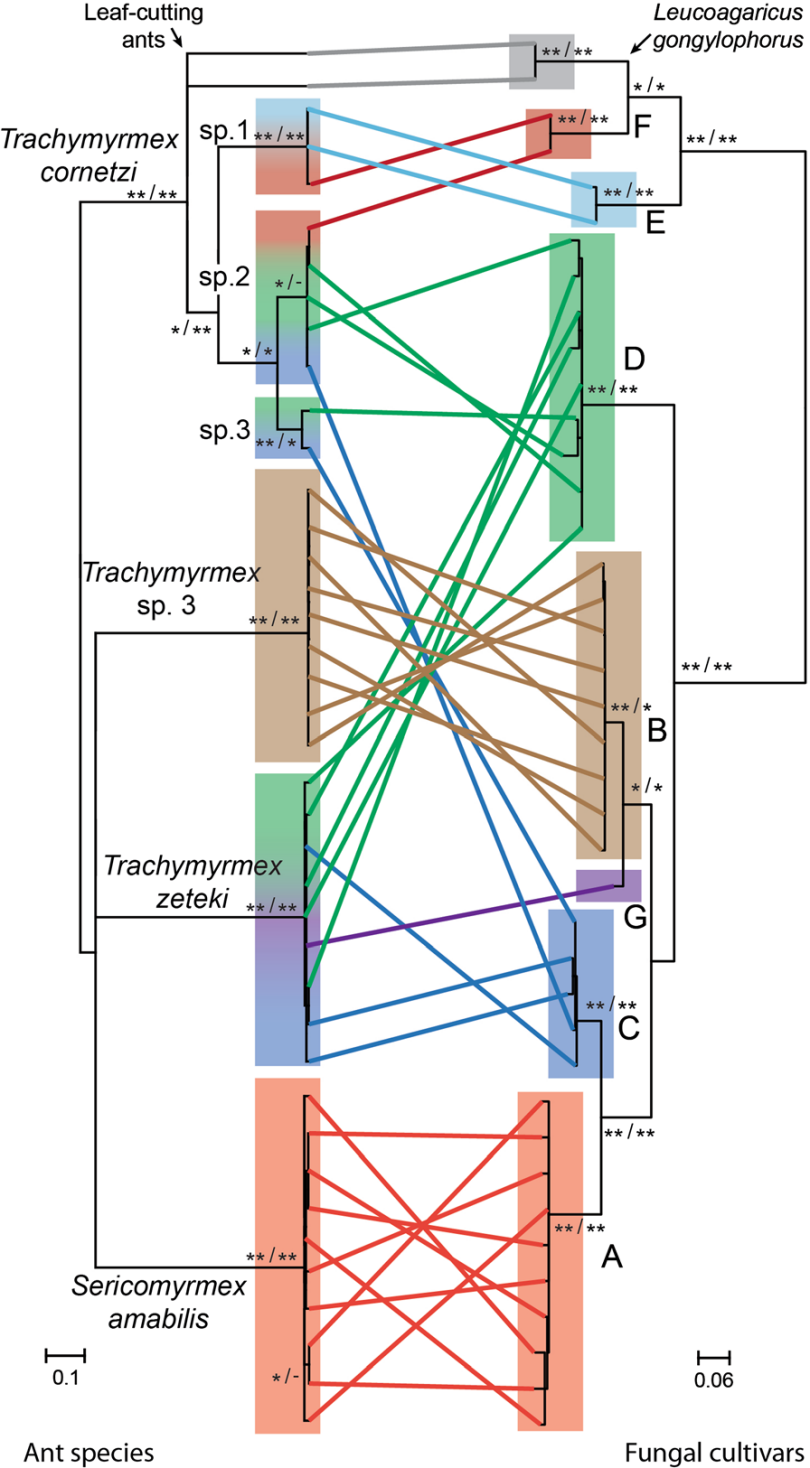
**Interaction specificity between Panamanian higher-attine ants and cultivars.** Fungus-growing ant and cultivar co-phylogeny color coded for each colony based on network analyses that independently identified groupings corresponding to seven fungal symbiont ITS haplotypes **(A-G)** and six ant species (*S. amabilis*, *T. zeteki*, *T.* sp. 3, and *T. cornetzi* sp. 1–3). *T. zeteki* and three *T. cornetzi* species (two of them cryptic and new to science) share a variable group of fungal ITS haplotypes **(C-G)**, whereas *S. amabilis* and *T.* sp. 3 each appear to cultivate a single ITS haplotype (**A** red and **B** brown, respectively). The phylogenies are based on maximum-likelihood and Bayesian analysis of a 549 bp alignment of partial CO1, tRNA, and CO2 ant sequences and a 740 bp alignment of ITS1, 5.8S, and ITS2 fungal sequences. The analyses included ant and cultivar sequences for the leaf-cutting ant species *Acromyrmex octospinosus* and *Atta cephalotes* (grey), which as expected from recent phylogenetic analyses branched off closer to the *T. cornetzi* lineage than the *Sericomyrmex/Trachymyrmex zeteki* clade [25]. Both trees are mid-point rooted and branch support is shown as: Maximum-likelihood (100 = **, 95 < *)/Bayesian (1.00 = **, 0.95 < *). Scale bars are substitutions per site.

Molecular analysis revealed distinct species-specific sequences for *T. zeteki, T.* sp. 3, and *S. amabilis*, but the 10 *T. cornetzi* colonies segregated in three groups based on a 95% maximum-likelihood posterior probability similarity cut-off, and thus likely represent distinct cryptic species (denoted *T. cornetzi* sp. 1–3, Figure 1). Network analysis recovered the exact same six groups of *Sericomyrmex* and *Trachymyrmex* fungus-growing ant species as in the phylogenetic analysis [see Additional file 2]. Phylogenetic analysis of the 38 identified fungal haplotypes produced seven distinct cultivar clades when using a 95% maximum-likelihood posterior probability similarity cut-off (labelled A-G; Figure 1) as previously applied in a similar analysis of cultivars of North American *Trachymyrmex* by Mikheyev et al. [32]. Also for the cultivars, network analysis identified the same haplotype groups and structured them in seven un-connected sub-networks with minimal variation within each network [see Additional file 2].

The sampled colonies of *T.* sp. 3 and *S. amabilis* cultivated a single genetically distinct fungal haplotype (A and B, respectively), whereas the four other *Trachymyrmex* species shared five fungal haplotypes (C-G), but to different degrees (Figure 1). The five *T. cornetzi* sp. 2 colonies and the nine *T. zeteki* had three, mostly but not entirely overlapping haplotypes each, and two fungal haplotypes (C and D) were associated with three different ant species (Figure 1). AMOVA of fungal haplotype distributions showed that sequence variation between ant species (39%) barely exceeded variation within ant species (34%) (Table 2). A second analysis excluding *S. amabilis* and *T.* sp. 3 because they had no cultivar variation showed that 83% of the fungal genetic variation occurred within species and only 17% across species, but this level did not quite reach statistical significance (Table 2). Fisher’s exact tests of contingency tables containing the same data confirmed a significantly non-random association pattern between ants and cultivars (*p* <0.001) for the full data set, but the null hypothesis of random association could no longer be rejected after excluding *S. amabilis* and *T.* sp. 3 and analyzing only the four ant species that cultivated more than a single cultivar haplotype (*p* =0.130).

**Table 2:** **AMOVA of intra- and interspecific cultivar variation**

**Source of variation**	**Degrees of freedom**	**Sum of squares**	**Variance component**	**explained variation (%)**	**Fixation index**	**P value**
**Complete dataset**						
Between genera	1	640.97	21.64	27	Φ_CT_ =0.27	0.3292
Among species within genera	4	774.25	31.87	39	Φ_ST_ =0.66	< 0.0001
Within species	32	876.26	27.38	34	Φ_SC_ =0.54	< 0.0001
Total	37	2291.48	80.89	100		
***T. cornetzi*** **sp. 1–3 and** ***T. zeteki***						
Among species within genera	3	330.22	12.18	17	Φ_ST_ =0.17	0.0645
Within species*	15	875.36	58.35	83		
Total	18	1205.58	70.53	100		

Results of AMOVA of *Leucoagaricus* symbiont diversity of the full dataset with three hierarchical levels and the second analysis considering only the *T. cornetzi* and *T. zeteki* species whose fungal symbionts were variable enough in their ITS sequences to represent different species (Figure 1). Significances are based on 10100 permutations evaluating whether fixation indices were different from a null distribution of variance parameters assuming samples were drawn from randomly chosen species.

*Only a single null distribution, assuming samples randomly drawn from within species, was generated, which precluded a permutation-based significance test for this level in the two-level pairwise AMOVA.

**Figure 2:**
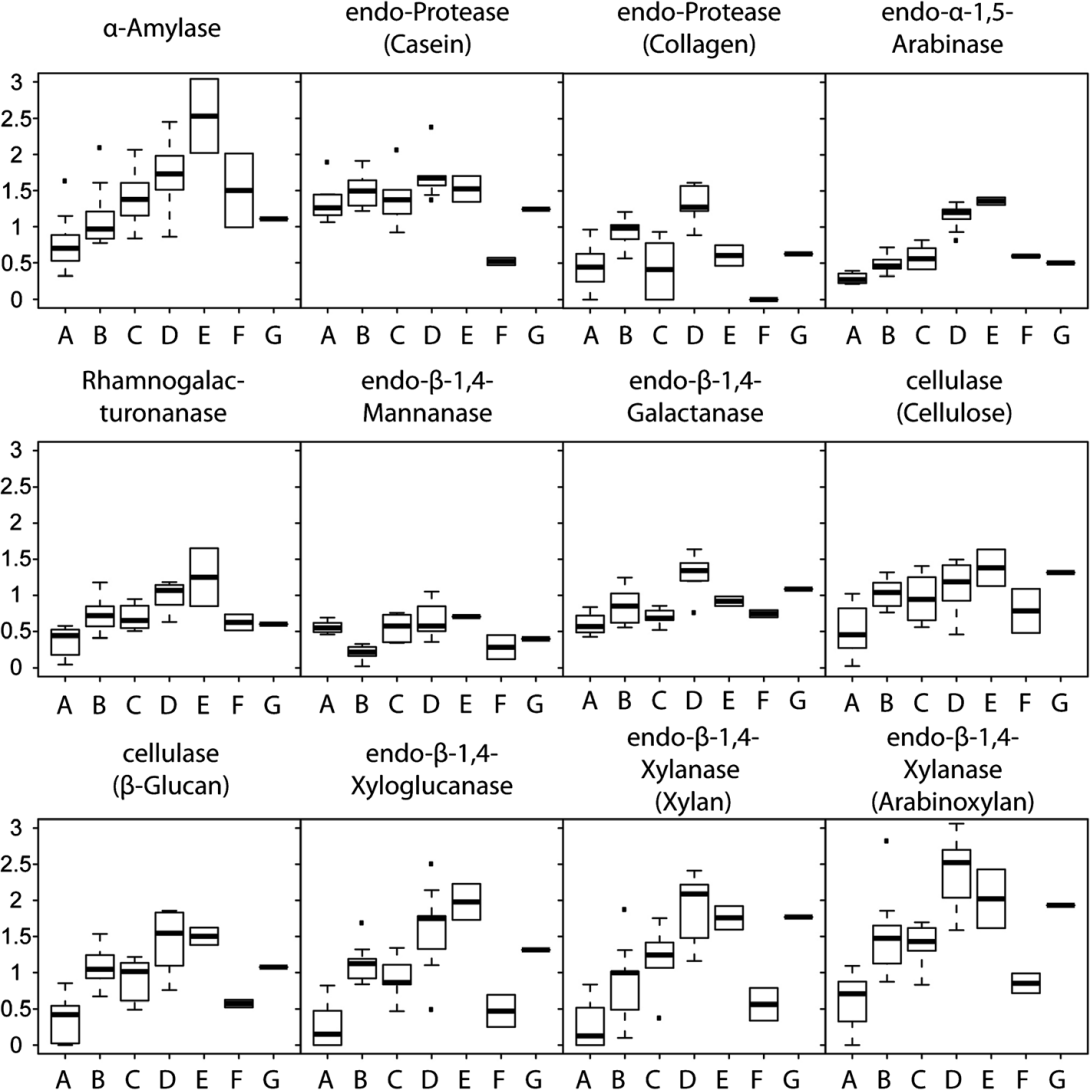
**Enzyme activity of cultivar haplotypes.** Activity of 12 carbohydrate active enzymes for the seven fungus garden ITS haplotypes **(A-G)** measured with an AZCL-plate assay with the Y-axis representing the diameter of the halo formed after a positive enzyme reaction. The spectra of 12 enzyme activities were significantly different between the fungus garden ITS haplotypes (see text for details). Boxplots represent the 1st and 3rd quartile with the median as an internal line, whiskers are the approximate 95% confidence intervals and individual dots are outliers.

**Figure 3:**
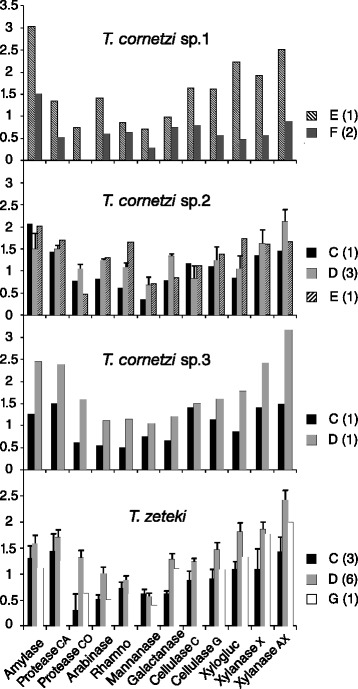
**Ant and cultivar specific enzyme activity.** Fungus garden ITS haplotype enzyme activities (diameter of the halo on AZCL plates after positive enzyme reactions) of 12 carbohydrate-active enzymes in four species of *Trachymyrmex* higher-attine ants. Sample size for each fungal ITS haplotype is given in brackets. Amylase: α-Amylase, Protease CA: endo-Protease (Casein substrate), Protease CO: endo-Protease (Collagen substrate), Arabinase: endo-1,5-α-Arabinase, Rhamno: Rhamnogalacturonanase, Mannanase: endo-β-1,4-Mannanase, Galactanase: endo-β-1,4-Galactanase, Cellulase C: Cellulase (cellulose substrate), Cellulase G: Cellulase (β-glucan substrate), Xylogluc: endo-β-1,4-Xyloglucanase, Xylanase X: endo-β-1,4-Xylanse (xylan substrate), Xylanase AX: endo-β-1,4-Xylanase (arabinoxylan substrate).

Activities of the 12 carbohydrate active enzymes differed significantly between the seven fungal haplotypes (Figure 2). The main enzyme and haplotype effects were both significant (F_11,372_ = 34.4, p <0.0001, F_6,372_ = 85.7, p <0.0001, respectively) and a significant interaction term showed that different enzymes were most active in different fungal haplotypes (F_66,372_ = 3.3, p <0.0001). The enzyme main effect is not meaningful, as the units of activity are not comparable across enzymes, but this ANOVA setup allowed us to partial out these overall activity level effects, so that the haplotype and interaction effects would be meaningful. Including ant species as an additional main factor allowed us to extend the analysis to a full three-way ANOVA for four ant species (Figure 3). This recovered the significant main effects of enzyme and fungal haplotype (F_11,108_ = 22.7, p <0.0001, F_4,108_ = 37.6, p <0.0001, respectively) and showed that farming ant species also significantly affected overall enzyme expression levels (F_3,312_ = 4.7, p =0.0038). The haplotype x enzyme interaction term was again significant (F_44,108_ = 1.6, p =0.0332), and we also obtained a significant haplotype × ant interaction term (F_2,108_ = 5.2, p =0.0072), whereas enzyme × ant interaction and the three-way ant × haplotype × enzyme interaction term were not significant (F_33,108_ = 0.8, p =0.7051, F_22,108_ = 0.5, p =0.9497, respectively).

## Discussion

Our analysis of the diversity of fungal cultivars among Panamanian higher-attine ants identified variable interaction specificity ranging from mutually high (one-to-one) degrees of species-specificity to low (many-to-many) specificity (Figure 1). Based on 10 and 9 samples, respectively, both *S. amabilis* and *T.* sp. 3 appeared to exclusively cultivate a single haplotype in the sampled population. The variation in our genetic markers and the statistical power of our analyses were sufficient to expose these differences between the common *Trachymyrmex* and *Sericomyrmex* species at our study site. We realize that replication of our type of study across multiple geographically distant sites in Central America would likely reveal interesting patterns of larger-scale diversity across populations, with some ant-fungus combinations being geographically conserved and others showing gradients, but studies of that kind would require an order of magnitude more effort to segregate local and regional/continental diversity in interaction specificity.

The results that we obtained can be interpreted as representing varying levels of co-evolutionary specialization, either to specific microhabitats or types of forage collected by the farming ants, or both. Haplotype A cultivated by *S. amabilis* generally had lower enzyme activity spectra compared to the haplotypes of the *Trachymyrmex* species except for *endo*-β-1,4-Mannanase (Figure 2). Samples of several *Sericomyrmex* species would have been needed to draw functional conclusions at the ant genus-level, but the reduced activity of especially *endo*-β-1,4-Xylanases that degrade rigid plant cell wall polymers suggests that *S. amabilis* cultivars may be less well adapted to handling recalcitrant plant forage material than *Trachymyrmex* species, consistent with *Sericomyrmex amabilis* having a somewhat larger proportion of fruits and berries in their typical forage spectrum at the same Panamanian site [34]. Larger scale geographic sampling of *Sericomyrmex* species would also be desirable to further test this contention.

In contrast to *S. amabilis,* the *T. zeteki* and *T. cornetzi* species in this Panamanian population were associated with several fungal strains that are shared between species (Figure 1, Table 2). All these species were fully sympatric in the Gamboa area although *T. zeteki* colonies were almost exclusively found on rather vertical soil surfaces at the base of trees and in stream banks, whereas *S. amabilis, T.* sp. 3 and the *T. cornetzi* species are widespread on the forest floor. Local microhabitat differences thus appear to be unimportant for fungal cultivar specialization, although it was notable that *T. cornetzi* species 2 and 3 essentially shared all symbionts with *T. zeteki*, but that *T. cornetzi* species 1 seemed to rear a different set of fungi more closely related to the leaf-cutting ant cultivar *Leucoagaricus gongylophorus* (Figure 1). Sample sizes were too small to draw any firm conclusions on cultivar specificity among the three cryptic *T. cornetzi* species, because one analysis showed that 83% of the overall variance in cultivar identity among ant species rearing more than a single cultivar occurred within species and a subsequent contingency table analysis failed to reject the hypothesis that associations are random within this group of four *Trachymyrmex* species.

Despite the obligate dependency of all higher-attine ants on specialized fungal cultivars, both parties may benefit from occasional “species-recombination” events to obtain better partnerships. Such novel ant-fungus relationships may be generated by occasional horizontal transmission of fungal cultivars between ant nests [27]. *T. zeteki*, the *T. cornetzi* species, and *S. amabilis* almost certainly have overlapping foraging territories so that founding queens that have lost their own cultivar may encounter burrows of con- or allo-specific other queens to steal gardens [28,58]. This implies that the two cases of one-to-one specialization in our data set (*T*. sp. 3 and *S. amabilis*) are unlikely to be due to lack of opportunity in encountering alternative symbionts. However, that leaves the question why their single lineages of symbionts did not diversify. Both ant species are common in the Panama canal zone [38,59,60], appear to have outbred panmictic mating systems, and there is no indication of population substructuring in one of them (*S. amabilis*) that was barcoded at multiple Panamanian sites (J. Liberti *et al.* unpublished results). The cultivation of a single fungal symbiont by *T.* sp. 3. and *S. amabilis* thus seems unlikely to be due to recent invasions accompanied by genetic bottlenecks, but larger-scale barcoding studies as discussed above will be needed to assess the degree to which these one-to-one interaction-specificities are maintained across Panama.

As far as interaction-specificity studies have been done in the lower attine ants, they also found the entire range of high to no cultivar specialization: *Mycocepurus smithii* is known to cultivate at least nine different symbionts in sympatry [61] but *Cyphomyrmex* species cultivate one symbiont per ant species throughout an entire lineage [23]. However, in the latter study, spatial sampling scales were much larger and replicate sampling within sites much less than our present single site approach. The lower attine ants rear non-specialized fungal symbionts that likely continue to exchange genes with free-living relatives at some low frequency [61], which may suggest that interaction specificity in the lower attine ants is as variable as in the higher attine ants, in spite of fundamental differences in symbiotic commitment between obligate and non-specialized crop symbionts. This underlines that the highly derived symbiont species *L. gongylophorus* that all *Atta* and *Acromyrmex* leaf-cutting ants rear may represent a unique form of specificity that came about during a secondary selective sweep only a few million years ago [29,31].

### Possible fitness consequences of cultivar genotype diversity

The interdependency of partners in the obligate higher-attine mutualism implies that natural selection is partitioned at two levels: selection acting on each of the individual partners and higher-level selection acting on the combined mutualistic entities [62,63]. However, the fact that ant colonies and garden symbionts are likely to commit for life (also after occasional horizontal swaps) implies that the group-level component is more important than the individual component, because monoculture rearing largely if not completely precludes the emergence of traits that cheat on the mutualistic services of the partner species [64]. It thus seems reasonable to assume that all colonies that we collected represented well-functioning entities of ant forage-provisioning and induced garden enzyme-activity, consistent with ANOVAs showing that both ant-species and fungal species had significant overall effects on garden enzyme activity spectra. However, the interaction terms suggested that fungal haplotype is the most fundamental factor because: 1. Enzyme activity spectra were additionally affected by the combination of ant species and fungal species, and 2. Activity of certain enzymes varied more than activity of others depending on fungal species, but not ant species (Figure 3). These findings are consistent with fungal plant degrading enzyme activities having a direct influence on colony fitness [19,37,65,66] and with these activities being plastically adjusted to the forage material used to manure fungus gardens [34,36]. We are aware that some enzyme activity may have been due to bacterial activities in fungus gardens [19], but their share in the fungus garden biomass is so minor that this cannot have affected our main results (see [66] for a more elaborate rationale of ignoring additional garden symbionts when interpreting overall enzyme activity spectra).

The capacity for carbohydrate active enzyme production is highly conserved among the basidiomycete fungi even though these enzymes are not constitutively present and only produced when induced by suitable substrate for degradation [67]. In the attine ant mutualism this induction has been outsourced to the farming ants that provide the substrate, mix it with fecal enzymes [34,56] and likely manage its addition to the actively growing garden parts in manners that imply rather specific enzyme activity induction [36]. Given this advanced form of mutual dependence, it thus seems reasonable to assume that differences in enzyme activity between gardens maintained by ant colonies within a small geographical area are either fungal-haplotype-specific or ant-specific or both, as we report in our present study (Figure 3). However, finding persistent performance variation across fungal cultivar species in four out of the six attine species that we studied appears to offer a conundrum because evolutionary models of mutualism stability tend to predict fixation of the most beneficial partner in a population [12]. Interaction specificities in our study populations of *Sericomyrmex amabilis* and *Trachymyrmex* sp. 3 were consistent with this expectation, but the four other *Trachymyrmex* species shared garden symbionts, were observed to associate with several of them and inferred statistically to perhaps even associate in a fairly random manner. This may reflect evolutionary tradeoffs between enzyme activity and other key traits such as desiccation tolerance, disease susceptibility or temperature sensitivity [10,68] that formal models have not yet considered (but see [69]). The selection regimes imposed by such trade-offs may also vary over time, particularly in geographic mosaics or meta-populations where negative frequency dependent selection on local adaptation may occur [10,11].

## Conclusions

Our results suggest that it is of crucial importance to keep confronting model predictions with detailed data sets. They also show that it is important that all cryptic species are identified, so that estimates of interaction specificity are both precise (free of unnecessary noise) and accurate (free of bias when there are cryptic species in one type of partner and not in the other). Our single-site study shows how objectives like this can be achieved, and how they can serve as modules in geographic sampling networks that have the potential to add explicit larger scale spatial components to studies of mutualistic interaction specificity. Once precise data on interaction specificity are available for a single representative site, a large number of interesting follow-up questions emerge: Are bilaterally specialized interactions restricted to more distinct microhabitat patches? Can relatively unspecialized host-symbiont interactions be subdivided in lineages that specialize on predictable fractions of the total niche space available? If so, would such assortment patterns be more likely to be driven by asexual fungal strains than by ant genotypes that recombine every generation? Would interactions that are specific at one site also tend to be specific at another geographically remote site and if so, would this likely involve the same two partners? Monoculture fungus farming by single ant colonies offers ideal possibilities to answer some of these questions, as most other mutualistic symbioses have the complication that hosts may either associate with several strains at the same time, or change partnership during their life-time [4,5].

## Availability of supporting data

The data set supporting the results of this article is included within the article (and its additional files).

## Additional files

Additional file 1:
**Enzyme activity measurements for the 38 samples colonies.**
Additional file 2:
**Detailed results of network analysis.**
