# Supplementary material for: Variable interaction specificity and symbiont performance in Panamanian Trachymyrmex and Sericomyrmex fungus-growing ants
Source: BMC Evol Biol. 2014 Dec 4;14:244. doi: 10.1186/s12862-014-0244-6 (PMC4262973; doi:10.1186/s12862-014-0244-6)
Supplement: Additional file 2: — Detailed results of network analysis. [file 12862_2014_244_MOESM2_ESM.pdf]

**Network analysis results**
Relevant output from TCS v. 1.21 analysis

## Ant Networks

```
PARSIMONY PROBABILITY
For 1 step(s),      P(90%) = 0.9993189000594782
For 2 step(s),      P(90%) = 0.9974565799863522
For 3 step(s),      P(90%) = 0.9947317351868009
For 4 step(s),      P(90%) = 0.9911062827467767
For 5 step(s),      P(90%) = 0.9865849716570294
For 6 step(s),      P(90%) = 0.9811775162679158
For 7 step(s),      P(90%) = 0.9748957304418876
For 8 step(s),      P(90%) = 0.9677537854640341
For 9 step(s),      P(90%) = 0.9597681955032399
For 10 step(s),     P(90%) = 0.9509577530083807
For 11 step(s),     P(90%) = 0.9413434557124126
For 12 step(s),     P(90%) = 0.9309484239440748
For 13 step(s),     P(90%) = 0.9197978082486945
For 14 step(s),     P(90%) = 0.9079186878391192
For 15 step(s),     P(90%) = 0.8953399604523783


RUN SETTINGS
Calculated maximum connection steps at 90% = 14
Gaps treated as fifth state

HAPLOTYPES
Number of haplotypes = 27

Haplotype list:
 - Tsp1_6      : Tsp1_4
 - Tsp1_3      : Tsp1_5
 - Tsp1_7      :
 - Tsp1_11     :
 - Tsp1_13     :
 - Tsp1_8      :
 - Tsp1_10     :
 - Tsp1_12     :
 - Sam2        : Sam5
 - Sam3        :
 - Sam1        : Sam6
 - Sam4        : Sam8
 - Sam7        :
 - Sam9        :
 - Sam10       :
 - Tsp3_8      : Tsp3_13 Tsp3_14 Tsp3_12 Tsp3_7 Tsp3_9
 - Tsp3_10     :
 - Tsp3_11     :
 - Aocto       :
```

```
 - Acephal     :
 - Tsp4_9      : Tsp4_10 Tsp4_16
 - Tsp4_19     : Tsp4_17
 - Tsp4_18     :
 - Tsp4_13     :
 - Tsp4_12     :
 - Tsp4_15     :
```

**OUTGROUP WEIGHTS**

*** Network 1 - *T. cornetzi* sp. 2
Tsp1_7     weigth = 0.14285714285714285
Tsp1_6     weigth = 0.5714285714285714
Tsp1_3     weigth = 0.2857142857142857
Total weight = 3.5
Biggest outgroup probability is Tsp1_6
(0.5714285714285714)

*** Network 2 - *T. cornetzi* sp. 3
Tsp1_11    weigth = 0.5
Tsp1_13    weigth = 0.5
Total weight = 1.0
Biggest outgroup probability is Tsp1_11    (0.5)

*** Network 3 - *T. cornetzi* sp. 1
Tsp1_12    weigth = 0.16666666666666666
Tsp1_8     weigth = 0.6666666666666666
Tsp1_10    weigth = 0.16666666666666666
Total weight = 3.0
Biggest outgroup probability is Tsp1_8
(0.6666666666666666)

*** Network 4 - *Sericomyrmex amabilis*
Sam4       weigth = 0.2608695652173913
Sam7       weigth = 0.043478260869565216
Sam10      weigth = 0.043478260869565216
Sam9       weigth = 0.043478260869565216
Sam1       weigth = 0.08695652173913043
Sam2       weigth = 0.2608695652173913
Sam3       weigth = 0.2608695652173913
Total weight = 11.5
Biggest outgroup probability is Sam4
(0.2608695652173913)

*** Network 5 - *Trachymyrmex* sp. 3
Tsp3_11    weigth = 0.05555555555555555
Tsp3_8     weigth = 0.8888888888888888
Tsp3_10    weigth = 0.05555555555555555
Total weight = 9.0
Biggest outgroup probability is Tsp3_8
(0.8888888888888888)

```
*** Network 6 - Acromyrmex octospinosus
Aocto      weigth = 1.0
Total weight = 1.0
Biggest outgroup probability is Aocto      (1.0)

*** Network 7 - Atta cephalotes
Acephal    weigth = 1.0
Total weight = 1.0
Biggest outgroup probability is Acephal    (1.0)

*** Network 8 - Trachymyrmex zeteki
Tsp4_13    weigth = 0.05555555555555555
Tsp4_18    weigth = 0.05555555555555555
Tsp4_9     weigth = 0.4444444444444444
Tsp4_19    weigth = 0.3333333333333333
Tsp4_15    weigth = 0.05555555555555555
Tsp4_12    weigth = 0.05555555555555555
Total weight = 9.0
Biggest outgroup probability is Tsp4_9
(0.4444444444444444)
```

# Fungus Networks

```
PARSIMONY PROBABILITY
For 1 step(s),      P(90%) = 0.9995449982421768
For 2 step(s),      P(90%) = 0.9981351488585823
For 3 step(s),      P(90%) = 0.9961097618736373
For 4 step(s),      P(90%) = 0.9934174087409977
For 5 step(s),      P(90%) = 0.9900569565895634
For 6 step(s),      P(90%) = 0.9860342715838435
For 7 step(s),      P(90%) = 0.9813559773087801
For 8 step(s),      P(90%) = 0.9760298946915947
For 9 step(s),      P(90%) = 0.9700651607333624
For 10 step(s),     P(90%) = 0.9634721986474911
For 11 step(s),     P(90%) = 0.9562626853327999
For 12 step(s),     P(90%) = 0.9484495172941622
For 13 step(s),     P(90%) = 0.9400467724843066
For 14 step(s),     P(90%) = 0.9310696681317402
For 15 step(s),     P(90%) = 0.9215345147734416
For 16 step(s),     P(90%) = 0.9114586666886794
For 17 step(s),     P(90%) = 0.900860468944134
For 18 step(s),     P(90%) = 0.8897592012770762


RUN SETTINGS
Calculated maximum connection steps at 90% = 17
Gaps treated as fifth state


HAPLOTYPES
Number of haplotypes = 34

Haplotype list:
 - Sam1        :
 - Sam9        :
 - Sam7        :
 - Sam2        :
 - Sam8        :
 - Sam6        :
 - Sam4        :
 - Sam10       :
 - Sam5        :
 - Sam3        :
 - Tsp1_13     :
 - Tsp4_19     :
 - Tsp1_7      :
 - Tsp4_17     :
 - Tsp4_18     :
 - Tsp3_13     :
 - Tsp3_14     : Tsp3_7 Tsp3_15 Tsp3_9 Tsp3_10 Tsp3_8
 - Tsp3_11     :
 - Tsp3_12     :
```

```
 - Tsp4_15    :
 - Tsp1_6     :
 - Tsp4_9     :
 - Tsp4_16    :
 - Tsp4_12    :
 - Tsp1_11    :
 - Tsp1_4     :
 - Tsp1_5     :
 - Tsp4_13    :
 - Tsp4_10    :
 - Acephal    :
 - Aocto      :
 - Tsp1_12    :
 - Tsp1_8     :
 - Tsp1_3     : Tsp1_10
```

**OUTGROUP WEIGHTS**

```
*** Network 1 - Cultivar group A
Sam5       weigth = 0.08333333333333333
Sam10      weigth = 0.08333333333333333
Sam6       weigth = 0.08333333333333333
Sam8       weigth = 0.08333333333333333
Sam2       weigth = 0.08333333333333333
Sam7       weigth = 0.08333333333333333
Sam9       weigth = 0.08333333333333333
Sam1       weigth = 0.08333333333333333
Sam4       weigth = 0.16666666666666666
Sam3       weigth = 0.16666666666666666
Total weight = 6.0
Biggest outgroup probability is Sam4 (0.16666666666666666)

*** Network 2 - Cultivar group C
Tsp4_18    weigth = 0.2
Tsp1_7     weigth = 0.2
Tsp4_19    weigth = 0.2
Tsp1_13    weigth = 0.2
Tsp4_17    weigth = 0.2
Total weight = 2.5
Biggest outgroup probability is Tsp4_18 (0.2)

*** Network 3 - Cultivar group B
Tsp3_12    weigth = 0.03125
Tsp3_13    weigth = 0.03125
Tsp3_14    weigth = 0.5
Tsp3_11    weigth = 0.4375
Total weight = 16.0
Biggest outgroup probability is Tsp3_14 (0.5)

*** Network 4 - Cultivar group G
Tsp4_15    weigth = 1.0
Total weight = 1.0
Biggest outgroup probability is Tsp4_15 (1.0)
```

```
*** Network 5 - Cutlivar group D
Tsp4_10    weigth = 0.058823529411764705
Tsp1_5     weigth = 0.058823529411764705
Tsp1_4     weigth = 0.058823529411764705
Tsp1_11    weigth = 0.11764705882352941
Tsp4_12    weigth = 0.058823529411764705
Tsp1_6     weigth = 0.058823529411764705
Tsp4_9     weigth = 0.11764705882352941
Tsp4_16    weigth = 0.23529411764705882
Tsp4_13    weigth = 0.23529411764705882
Total weight = 8.5
Biggest outgroup probability is Tsp4_16 (0.23529411764705882)
```

*** Network 6 - *Leucoagaricus gongylophorus*

```
Acephal    weigth = 0.5
Aocto      weigth = 0.5
Total weight = 1.0
Biggest outgroup probability is Acephal (0.5)

*** Network 7 - Cultivar group F
Tsp1_12    weigth = 0.5
Tsp1_8     weigth = 0.5
Total weight = 1.0
Biggest outgroup probability is Tsp1_12 (0.5)

*** Network 8 - Cultivar group E
Tsp1_3    weigth = 1.0
Total weight = 2.0
Biggest outgroup probability is Tsp1_3 (1.0)
```
